# Improving Registration and Dataflows Between Pediatric Oncology Clinics and the Childhood Cancer Registry of Switzerland: Protocol for SwissPedCancer Quality Assurance Study

**DOI:** 10.2196/87007

**Published:** 2026-03-10

**Authors:** Yara Shoman, Lorenz Leuenberger, Grit Sommer, Julia Anna Bielicki, Pierluigi Brazzola, Sophia della Valle, Manuel Diezi, Daniel Drozdov, Fabienne Gumy-Pause, Ana Guerreiro Stücklin, Ursula M Kuehnel, Katrin Scheinemann, Christina Schindera, Freimut Schilling, Nicolas Waespe, Ben D Spycher, Luregn J Schlapbach, Claudia E Kuehni, Fabiën N Belle, SwissPedHealth Consortium, Andrea Agostini

**Affiliations:** 1 Institute of Social and Preventive Medicine, University of Bern, Mittelstrasse 43, Bern, CH-3012, Switzerland, 41 (0)31 631 35 07; 2 Childhood Cancer Registry of Switzerland (ChCR), Bern, Switzerland; 3 Graduate School for Health Sciences, University of Bern, Bern, Switzerland; 4 Department of Paediatric Infectious Diseases, University Children’s Hospital Basel, Basel, Switzerland; 5 Centre for Neonatal and Paediatric Infection, St George's University, London, United Kingdom; 6 Pediatric Institute of Italian Switzerland, Bellinzona, Bellinzona, Switzerland; 7 Lausanne University Hospital (CHUV), University of Lausanne, Lausanne, Switzerland; 8 Division of Pediatric Hematology Oncology, Children’s Hospital, Kantonsspital Aarau (KSA), Aarau, Switzerland; 9 Division of Pediatric Oncology and Hematology, Department of Women, Child and Adolescent, Geneva University Hospitals (HUG), Geneva, Switzerland; 10 CANSEARCH Research Platform for Pediatric Oncology and Hematology, Department of Pediatrics, Gynecology and Obstetrics, Faculty of Medicine, University of Geneva, Geneva, Switzerland; 11 Division of Oncology and Children's Research Center, University Children's Hospital of Zurich, Zurich, Switzerland; 12 Division of Hematology/Oncology, Children’s Hospital of Eastern Switzerland, St. Gallen, Switzerland; 13 Faculty of Health Sciences and Medicine, University of Lucerne, Lucerne, Switzerland; 14 Depaertment of Pediatric Oncology/Hematology, University Children’s Hospital Basel, Basel, Switzerland; 15 Children’s Hospital of Central Switzerland, Lucerne, Switzerland; 16 Division of Pediatric Hematology and Oncology, Department of Pediatrics, Inselspital, Bern University Hospital, University of Bern, Bern, Switzerland; 17 Division of Intensive Care Oncology and Neonatology, and Children's Research Center, University Children’s Hospital Zurich, University of Zurich, Zurich, Switzerland; 18 Child Health Research Centre, The University of Queensland, Brisbane, Queensland, Australia; 19 See Acknowledgments

**Keywords:** cancer registration, cancer diagnosis, case ascertainment, chemotherapy, data quality, radiotherapy, Europe

## Abstract

**Background:**

Cancer registries are essential to monitor cancer incidence and survival to provide better quality cancer data for research. In Switzerland, the pediatric oncology units within pediatric hospitals actively report cancer cases, and the coding and registration team of the Childhood Cancer Registry (ChCR) enters data manually from medical files into the registry database. There are no automated data transfers or feedback loops between the pediatric oncology clinics and the ChCR. This ongoing process is time-consuming, inefficient, and a source of potential errors.

**Objective:**

SwissPedCancer aims to explore the options for automated data transfers from clinical data warehouses and feedback loops to make cancer registry processes more efficient.

**Methods:**

SwissPedCancer is a nested project within the national data stream initiative, the Swiss Pediatric Personalized Research Network (SwissPedHealth). Since September 2022, SwissPedHealth has developed and piloted structures to make routine clinical data from pediatric oncology clinics available for monitoring, benchmarking, and research in an interoperable, standardized, and quality-controlled way. SwissPedCancer expects to include approximately 2800 patients diagnosed with cancer before the age of 20 years between 2017 and 2023. The pediatric oncology clinics’ data and the manually validated ChCR data will be delivered separately to a secure national computing network for health-related data (Biomedical Information Technology). We will compare these two data sources to assess completeness (case ascertainment), accuracy (validity), and timeliness of cancer registration in the ChCR. We will evaluate data on diagnosis, treatments, underlying genetic disease, remission, relapse, and late effects. SwissPedCancer will provide a framework for optimizing standardized and uniform data transfers between pediatric oncology clinics and the ChCR and for other registries within Switzerland.

**Results:**

The project was funded in September 2022 and received ethics exemption in October 2023. Data extraction from participating hospitals and the ChCR is expected to commence in January 2026. Study results are anticipated to be available in summer 2026.

**Conclusions:**

SwissPedCancer aims to reduce manual workload while improving the completeness, accuracy, timeliness, and comparability of childhood cancer data in Switzerland. The project will contribute to a robust, interoperable, and sustainable national infrastructure supporting high-quality cancer registration, timely analyses, and evidence-based decision-making.

## Introduction

Cancer is one of the leading causes of death in childhood and adolescence [1]. In Switzerland, approximately 400 new cancer cases are diagnosed every year in children and adolescents aged younger than 20 years [2]. The incidence rate in children or adolescents in Switzerland is comparable to that of neighboring countries [3,4]. According to the Childhood Cancer Registry of Switzerland (ChCR), the incidence rate of childhood cancer is approximately 108.2 cases per million children annually [5]. Cancer registries play a crucial role in understanding and addressing the burden of cancer among children and adolescents [6]. They enable the collection of standardized data on cancer incidence, prevalence, spectrum of diagnoses, mortality, survival, treatments, and the course of the disease [7]. The Swiss Childhood Cancer Registry was founded in 1976 by the Swiss Pediatric Oncology Group [8]. Before 2020, registration of childhood cancer cases was done on a voluntary basis by all pediatric oncology clinics but already with a high completeness and data quality for children aged less than 15 years [8-14]. Adolescents aged between 16 and 20 years are often treated at adult oncology clinics where registration was less systematic in the registry. On January 1, 2020, the Swiss Federal Act on the Registration of Cancerous Diseases (Cancer Registration Act [CRA]; RS 818.33) and its related Cancer Registration Ordinance (CRO) came into force, mandating the systematic, uniform, and comprehensive registration of all cancer cases across the country. The act aims to improve understanding of cancer patterns, support research, and enhance the quality of diagnosis, treatment, and care. Health care providers are legally obliged to report relevant cancer data to cantonal registries for adults and to the national ChCR for children and adolescents [15]. The ChCR supports evidence-based public health planning, ultimately contributing to better care and prognosis for children with cancer [2,16].

As part of the growing national and international efforts to standardize cancer registration data [17-19] and automate dataflows [20,21], SwissPedCancer represents a key initiative to improve the quality and efficiency of childhood cancer registration in Switzerland. SwissPedCancer is a nested project of the pediatric national data stream (NDS) initiative, the Swiss Pediatric Personalized Research Network (SwissPedHealth), which supports personalized health research through a harmonized dataset across Switzerland [22]. In line with global efforts in countries such as Sweden, Denmark, Norway, the United Kingdom, and the United States [17,19,20,23,24]—where integrated, interoperable systems allow for timely cancer data capture—SwissPedCancer seeks to modernize data transfers from Clinical Data Warehouses (CDWs) to the ChCR. Currently, hospital staff manually enter clinical information from medical files into the clinical information system of CDWs of reporting hospitals. Hospital-based clinical research associates send medical files of patients with cancer to the ChCR, using methods such as secure email or online transfer tools. This process varies across pediatric oncology clinics: It occurs at the time of diagnosis, monthly, or in even larger time intervals, and it can involve individual or multiple patient files. At the ChCR, trained staff enter and code personal and cancer-related data manually. These procedures entail a high manual workload, lead to duplication of work (entering data twice at the CDWs and ChCR) with considerable delays from event to registration, and increase the risk of errors. SwissPedCancer aims to harmonize and automate these dataflows, minimizing delays in patient registration, reducing redundancy in data entry, and enhancing data security by limiting the number of individuals handling sensitive health information. Automation could save time and resources and improve the accuracy, timeliness, and completeness of registry data. As the ChCR is Switzerland’s oldest national pediatric disease registry with high case completeness, the advancements achieved through SwissPedCancer could serve as a model for similar national and international registries.

SwissPedCancer aims to assess five quality indicators for cancer registration in the ChCR and improve dataflows between pediatric oncology clinics and the ChCR:

1. Assess adherence of cancer data to national and international standards: we will evaluate whether cancer data registered at the pediatric oncology clinics and at the ChCR align with the national and international guidelines for cancer registration and the standardization of practices.
2. Assess completeness of case ascertainment and case completeness at the ChCR from the nine pediatric clinics: we will evaluate if all eligible children with cancer, who should be registered according to the inclusion criteria of the ChCR, are registered within the ChCR dataset. We will evaluate how registered and unregistered cases differ and the reasons for variation:
  - by period, comparing the periods before and after the CRA came into force (January 1, 2020) when notification of cancer cases became compulsory for medical professionals and
  - by other potential predictors of registration, for example, hospital or medical subspecialty overseeing patients’ treatments, region in Switzerland, cantonal cancer registry catchment area, cancer type, and age group.
3. Assess data accuracy (validity): we will evaluate the quality of registered data in the ChCR by comparing data from CDWs and ChCR. Data accuracy relies on the quality of data entry in the clinical information systems, the fidelity of corresponding data in CDWs, and the level of proficiency in abstracting, coding, and recording, both at the pediatric oncology clinics and the ChCR level.
4. Assess timeliness: we will evaluate timeliness, defined as the time interval between the date of cancer diagnosis (date of incidence) and the date of inclusion in the registry and official cancer statistics.
5. Optimize the dataflow between pediatric oncology clinics, the CDWs, and the ChCR: we will develop standard operating procedures to improve registration, set up dataflows between CDWs and the ChCR, and develop feedback loops between pediatric oncology clinics and the ChCR.

## Methods

### Ethical Considerations

SwissPedCancer is a national audit and quality improvement project. SwissPedCancer has been exempted from the requirement to obtain ethical approval by the Cantonal Ethical Committee Bern (Req-2023‐01081); thus, individual informed consent was waived. All data used for this study were deidentified before analysis. Data access and handling complied with applicable Swiss data protection regulations, and appropriate technical and organizational measures were implemented to safeguard participant privacy and confidentiality (the Federal Act on the Registration of Cancerous Diseases in Switzerland, known as the CRA [15]. No financial or other compensation was provided to participants, as no direct participant contact or intervention occurred.

### Inclusion Criteria

SwissPedCancer will include data from inpatients and outpatients younger than 20 years at the time of cancer diagnosis who were treated at a Swiss pediatric oncology clinic: Kantonsspital Aarau, Aarau; Universitäts-Kinderspital beider Basel, Basel, Ente Ospedaliero Cantonale, Bellinzona; Inselspital, Bern, Hôpitaux Universitaires de Genève, Geneva; Centre hospitalier universitaire vaudois, Lausanne; Luzerner Kantonsspital, Luzern; Ostschweizer Kinderspital, St. Gallen; or Kinderspital Zürich, Zurich. SwissPedCancer will initially include patients diagnosed between January 1, 2017, and December 31, 2023. We will include patients diagnosed with malignant neoplasms and low-grade central nervous system tumors, excluding skin basalioma, according to *International Statistical Classification of Diseases, Tenth Revision* (*ICD-10*), to align with the ChCR inclusion criteria and the CRO (Table 1). SwissPedCancer is a nested study of the NDS SwissPedHealth, which has been described in detail elsewhere [22].

**Table 1.** Codes of cancer diagnoses registered in the Childhood Cancer Registry.

*ICD-10*^a^ codes	Notifiable cancers
C codes	
C00-C97	Malignant neoplasms
D codes	
D00-D03, D05-D09	Carcinoma in situ
D32, D33, D35(D35.2, D35.3, D35.4)	Benign neoplasms of the meninges, brain, and other parts of the central nervous system and benign neoplasms of endocrine glands of the head or brain (pituitary gland, epiphysis, ductus craniopharyngealis)
D37-D48	Neoplasms of uncertain or unknown behavior
D61	Unspecified aplastic anemia
D76	Other unspecified diseases involving lymphoreticular tissue and the reticulohistiocytic system

a *ICD-10*: *International Statistical Classification of Diseases, Tenth Revision*.

### Study Design

This is a retrospective comparative study that aims to compare information available in CDWs with the dataset of the national ChCR. We expect to include approximately 2800 patients from the nine pediatric oncology clinics for this period. This represents approximately 80% of the total childhood cancer cases in Switzerland [2,8,10-14,25,26].

### Data Sources

#### Childhood Cancer Registry of Switzerland

Swiss pediatric oncologists started in 1976 to nationally register children diagnosed with leukemia, lymphoma, central nervous system tumors, malignant solid tumors, or Langerhans cell histiocytosis to facilitate inclusion in and comparison with international studies. Initially, only data from patients enrolled in clinical trials were documented in the ChCR. Since 1981, the ChCR also included non–study participants [27,28]. In 2020, the legal framework changed, and the registry is owned by the Federal Government. Under the new law, medical professionals are legally obliged to notify childhood cancer cases to the ChCR [15]. The ChCR registers all children and adolescents with a primary diagnosis before 20 years of age and who belong to the permanent resident population of Switzerland. The ChCR records longitudinal data about the course of disease, treatments, and follow-up information. Patients or legal representatives have the right to object to the registration (veto). The ChCR analyzes the data to monitor the incidence and survival of cancer in children and adolescents, to use them for national health reporting, and to make data available for researchers [2]. The ChCR is aligned with international and national standards and uses the Swiss coding handbook [2]. It collects the following data: identifying data, diagnosis coded according to *ICD-10*, International Classification of Diseases for Oncology (ICD-O-3), and International Classification of Childhood Cancer (ICCC-3) codes, staging and grading, treatments, underlying genetic disease, progression, remission, relapse, late effects, prognostic factors, and study participation in clinical trials (Table 2) [8]. Since 2009, the ChCR published biannual reports [2,8,10-14,25,26,29].

**Table 2.** Transfer of data from routine clinical information of pediatric oncology clinics for the project SwissPedCancer and overlapping variables of the Childhood Cancer Registry (ChCR) in Switzerland.

SwissPedCancer CDWs^a^ data^b^	SwissPedCancer standard	Nonexhaustive list of overlapping ChCR variables	ChCR standard
Administrative case, care handling	—^c^	First treatment complex, treatment end date, accuracy of treatment end date, diagnostic institutions, treatment institution(s)	—
Administrative sex	SNOMED CT^d^	Sex	—
Age, birth	—	Date of birth, accuracy for date of birth	—
Data provider	UID^e^	Diagnostic institution(s), treatment institution, first treatment complex institution(s)	UID
Death	—	Vital status, date for vital status, accuracy for date of vital status	—
Drug administrative event	GTIN^f^, SNOMED CT	ATC^g^ code(s), standard drug combinations, start date of treatment, accuracy for start date of treatment, treatment end date, accuracy of treatment end date, study patient, type of study, study protocol, regimen, treatment institution, first treatment complex institution(s)	ATC
Drug prescription	SNOMED CT	Start date of treatment, accuracy for the start date of treatment, treatment end date, accuracy of treatment end date, study patient, type of study, study protocol, regimen, (first) treatment goal(s)	—
Diagnosis, billed diagnosis	*ICD-10*^h^, ICD-O-3^i^, SNOMED CT	*ICD* version, *ICD* code, inherited predispositions, type of medical condition, medical condition *ICD* version, medical condition *ICD* code, *ICD* version for causes of death, type of recurrence(s)/transformation(s), diagnostic institution(s)	*ICD-10*, ICD-O-3, ICCC-3^j^
Billed procedure	CHOP^k^	CHOP treatment code	CHOP
Health care encounter	—	Treatment end date, accuracy of treatment end date, diagnostic institution(s), treatment institution	—
Oncology diagnosis	ICD-O-3	ICD-O version, ICD Topography, ICD-O Morphology, ICD-O Behavior, ICD-O Histological grade, molecular or cytogenetic marker(s) tested, molecular or cytogenetic marker(s) test result, topography of metastases at diagnosis	ICD-O-3
CDW subject pseudo identifier	—	Study ID, NCID^l^	—
Nationality	ISO^m^ code (ISO 3166)	Nationality	—
Consent, study participation	—	Study patient, type of study, study protocol, regimen	—

a CDW: clinical data warehouse.

b CDW data are based on Swiss Personalized Health Network concepts. Swiss Personalized Health Network concepts are generalizable building blocks, which can be used in different contexts. Each concept contains all information necessary to understand it, and concepts can be combined to composed concepts, which again can be combined to more complex compositions [30].

c Not available.

d SNOMED CT: Systematized Nomenclature of Medicine Clinical Terms.

e UID: standardized business identification number.

f GTIN: Global Trade Item Number.

g ATC: Anatomical Therapeutic Chemical Classification.

h *ICD-10*: *International Statistical Classification of Diseases, Tenth Revision*.

i ICD-O-3: International Classification of Diseases for Oncology.

j ICCC: International Classification of Childhood Cancer.

k CHOP: diagnoses and the Swiss surgical classification.

l NCID: National Case Identifier.

m ISO: International Organization for Standardization.

#### Swiss Personalized Health Network: Data From CDWs

In Switzerland, the Swiss Personalized Health Network (SPHN) has built a shared standard infrastructure to make health-related data harmonized and interoperable, ease the work of data providers, and enhance the quality of routinely collected data [31]. SPHN aims to use and exchange health-related data between data providers and researchers in a FAIR (Findable, Accessible, Interoperable, and Reusable) manner [32]. SPHN supports the SwissPedHealth [22], a joint pediatric NDS initiative. The SwissPedHealth NDS will include data from the CDWs of all Swiss pediatric oncology clinics in a standardized interoperable format based on the Resource Description Framework [22,33]. SwissPedCancer is a nested project of the SwissPedHealth NDS initiative that aims to pilot this data infrastructure in the context of quality improvement (Table 2). This data structure incorporates existing ontologies to ensure standardized and interoperable data representation from all available CDW data, including data from pediatric and adult departments, inpatients, outpatients, and pathology reports [22].

### Data Protection

Health-related personal data in this project are pseudonymized for the research team (Table 3). Only ChCR registry staff members with the relevant legal mandate have access to identifying information. To enable patient linkage, the CDWs generate a subject-specific pseudo identifier for each patient, which serves as the sole means of identification during analysis. ChCR employees will link the subject pseudo identifier with the ChCR Study ID using the Swiss personal social security insurance number (Old-Age and Survivors’ Insurance [OASI] number) or, if not available, using names, sex, and date of birth. The process is multifaceted to satisfy all legal and data protection obligations.

**Table 3.** Summary of definitions of terms.

Term	Definition
ChCR^a^ team	Staff of the ChCR including employees in cancer registration, medical coders, and statisticians
SwissPedCancer team	Researchers working for the SwissPedCancer project at the Institute of Social and Preventive Medicine, University of Bern
ChCR study ID	ID given to the participants of this specific study. This ID differs from the ID used within the ChCR (PidN^b^), so researchers cannot identify patients
CDW^c^ subject pseudo identifier from pediatric oncology clinic	ID given to patients in the clinical data warehouse of a pediatric oncology clinic
PidN	ID given to patients at the ChCR

a ChCR: Childhood Cancer Registry.of Switzerland

b PidN: patient identification number.

c CDW: clinical data warehouse.

### Data Governance

We used the project-specific Data Project Consortium Agreements from the governance structure of SwissPedHealth, which consists of a steering level with an overarching Steering Committee, an Infrastructure Executive Board and a Scientific Advisory Board, a management level comprising the Executive Office (Executive Board and Management Office), and an operational level with work packages covering the data integration and research programs [22]. The Data Project Consortium Agreements of SwissPedCancer focus on project-specific governance aspects, including project aims, sponsorship, project partners, data needs, and timelines. Principles of data governance and regulatory compliance respect primary ownership of routine data by the hospitals with their respective CDWs and research-only data by the respective research teams. Requests to access data are governed accordingly at the level of the SwissPedHealth Data Access Request Committee of the SwissPedHealth Infrastructure Executive Board [22].

### Dataflows, Patient Linkage, and Data Storage

**Figure 1.**
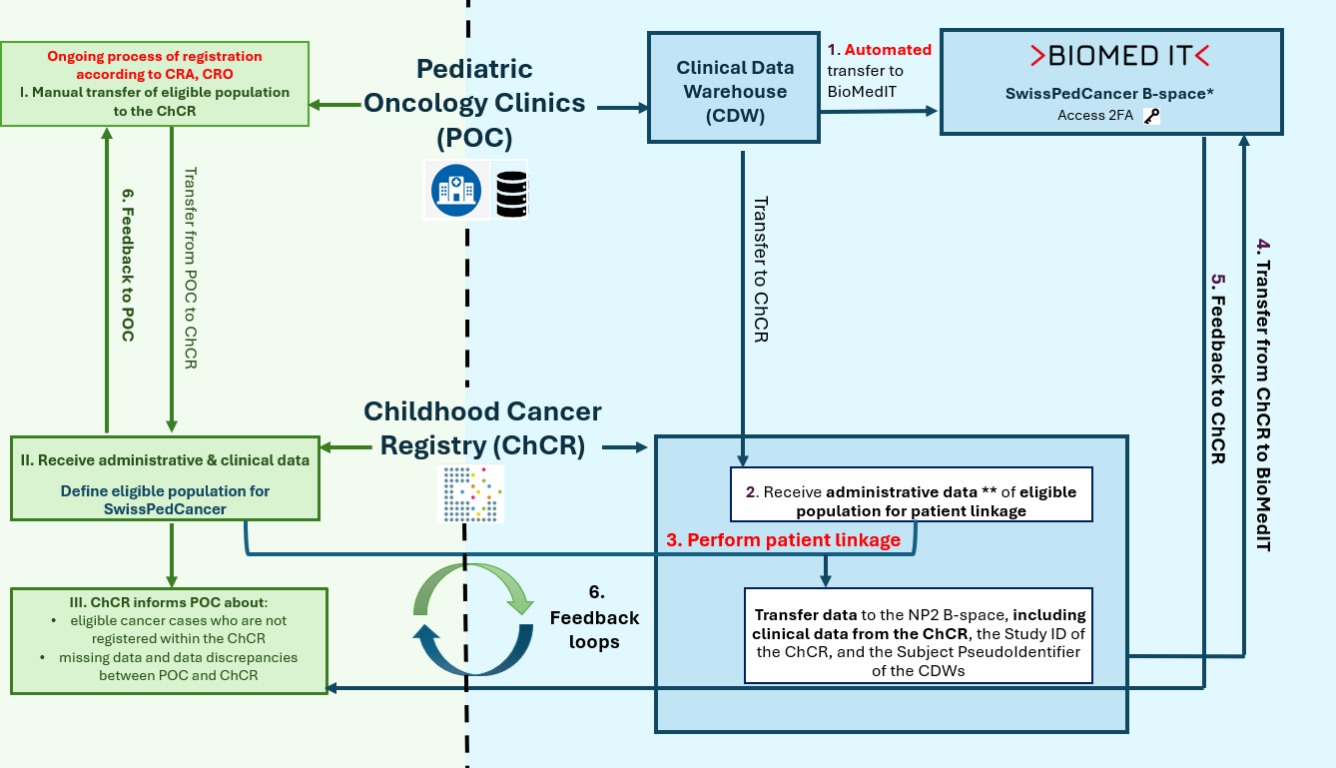
Dataflows within SwissPedCancer (steps 1‐5) and the ongoing process of cancer registration (steps I-III). *Merge the CDWs and ChCR datasets in the NP2 B-space of BioMedIT with the subject pseudoidentifier of the CDWs and the study ID of the ChCR: In the first stage, only ChCR employees have permission to access the SwissPedCancer B-space to erase those not eligible. Not eligible are non-Swiss residents, those who gave a veto against cancer registration, and those patients who initially had a suspicion of cancer but in whom cancer was excluded later. In the second stage, the SwissPedCancer research team can access the SwissPedCancer B-space. The dataset is now ready to assess quality assurance. **Administrative data: names, sex, date of birth, and Swiss personal social security insurance number (OASI number). In green color, ongoing process of registration at the Childhood Cancer Registry of Switzerland: data transfer from pediatric oncology clinics to ChCR. In blue color, SwissPedCancer processes: automated transfers from CDWs of hospitals to ChCR. BioMedIT: Biomedical Information Technology; CRA: Cancer Registration Act; CRO: Cancer Registration Ordinance.

The dataflows within SwissPedCancer are divided into several phases (Figure 1).

1. Automated transfers from CDWs to Biomedical Information Technology (BioMedIT): The CDWs of each pediatric oncology clinic will identify eligible patients (Table 1). *Selection of eligible population*: Pediatric oncology clinics will identify eligible patients in their CDW in line with the CRA and CRO [15] (Table 1). The CDWs will convert clinical data to SPHN standards and the Resource Description Framework format [34]. The CDWs will encrypt and transfer two datasets (Table 2) for the SwissPedCancer project. The *first dataset* is the full dataset of *clinical data*, which is transferred to the secure SwissPedHealth project space, so-called “NDS B-space” on the BioMedIT platform [35]. Clinical data include information on diagnosis (*ICD-10*, ICD-O-3), medical procedures, and death status. The NDS B-space serves as a data repository where data quality assessments and remediations are performed. After quality assessments by the central data manager of the NDS B-space, data are filtered and transferred to the SwissPedCancer project-specific B-space, so-called “workspace” [22].
2. Administrative data of eligible population: The second dataset is a minimal administrative dataset, which is transferred to the ChCR, including the subject-specific pseudo identifier, OASI number, name, sex, and date of birth for patient linkage.
3. Patient linkage within the ChCR: The ChCR team will perform a one-time patient linkage between the minimal administrative dataset from the CDWs and the dataset from the ChCR. After patient linkage, the ChCR will prepare a CSV file with the subject pseudo identifier of the pediatric oncology clinics, the ChCR Study ID, and all ChCR clinical data with overlapping information with CDW variables (Tables 2 and 3). ChCR staff will pseudonymize this dataset before transferring it to BioMedIT by deleting identifying information such as OASI numbers or names.
4. Data transfer from ChCR to BioMedIT: ChCR staff will transfer the pseudonymized dataset to BioMed IT. The data preparation in the BioMed IT workspace will contain two stages. Logging in to this secure workspace requires 2-factor authentication, and access to data is only possible via trusted networks. In the first stage, only ChCR employees have access to the workspace. ChCR employees will combine the pseudonymized ChCR dataset with the full dataset from the CDWs. According to the CRA and CRO, only ChCR staff are allowed to handle the vetoes against cancer registration. As the ChCR is not allowed to share the identity of patients who submitted a veto against cancer registration—only patients with cancer and their legal representatives hold this right—ChCR employees will exclude all cases at the time of diagnosis who are not eligible. In summary, not eligible are non-Swiss residents, patients who vetoed against cancer registration, and patients with an initial cancer suspicion that was later ruled out. In the second stage, the SwissPedCancer research team will obtain access to the dataset from the ChCR research team after the exclusion of noneligible cases; the dataset is now ready for analysis.
5. Feedback to the ChCR, data evaluation: The SwissPedCancer team will evaluate data completeness and accuracy. The SwissPedCancer team will share their findings with the ChCR, the pediatric oncology clinics, and the CDWs on, for example, missing cases, missing data, or discrepancies, to improve data quality.
6. Feedback to pediatric oncology clinics to improve cancer registration according to CRA: The ChCR will contact the pediatric oncology clinics to request a defined dataset for eligible children who have not yet been reported to the registry.

### Data Analysis Plan

SwissPedCancer will assess five quality indicators for cancer registration in the ChCR, proposed by the International Agency for Research on Cancer (IARC) and by the National Agency for Cancer Registration (NACR) [36,37]: (1) adherence of the data to national and international standards, (2) completeness of case ascertainment, (3) case completeness, (4) accuracy or validity of the data, and (5) timeliness of cancer registration.

#### Aim 1: Adherence of Registered Data to the National and International Standards

We will evaluate the extent to which data follow the national (NACR) standards and the international (IARC) recommendations [36,37]. We will assess how standard terminologies such as the Systematized Nomenclature of Medicine Clinical Terms (SNOMED CT), *ICD-10*, and ICD-O-3 are used across data sources, examining whether key variables are coded consistently. This evaluation will allow us to determine the interoperability of the datasets and their readiness for national and international comparisons.

#### Aim 2: Completeness of Case Ascertainment and Case Completeness

We will focus on evaluating the completeness of case ascertainment and case completeness in a multidimensional approach. To achieve this, we will compare patient data recorded during clinical care in pediatric oncology clinics to corresponding records in the ChCR. This includes diagnosis, presence of metastases, treatment details, genetic predisposition, remission status, relapse events, and late effects. Various computational methods [38] will identify missing cases and assess the extent and characteristics of incomplete data. For the assessment of completeness of case ascertainment and case completeness, we will consider the CDWs as the standard reference because they are the source of data.

We will conduct the following assessments:

1. Quantitative completeness of case ascertainment and case completeness to see whether all reportable cases are registered in the ChCR and if all variables for each case are captured in the ChCR.
2. Qualitative or semiquantitative completeness to reflect the thoroughness of each case record captured in the CDWs. We will assess the percentage and importance of missing variables or information of variables (eg, for diagnosis, are all codes available, eg, *ICD-10*, ICD-O-3, ICCC-3; Table 2). The completeness checks will include:
  - Variable presence: we will check the completeness of the extracted CDW data and the ChCR dataset, for example, if all variables listed in Table 2 are present and captured in both datasets. For example, apart from treatment received, the start and end date of treatment, the cumulative doses, and the location (body part) of radiotherapy are also captured in both datasets.
  - Data variable agreement: we will compare CDW data to ChCR data to evaluate if information is the same or compatible (eg, diagnosis, according to ICD-10 and ICD-O-3 codes, or treatment).
3. Temporal completeness: we will evaluate how completeness evolves over time, particularly comparing periods before and after the implementation of the CRA and CRO [39].

#### Aim 3: Data Accuracy (Validity)

The third objective is to assess the accuracy (validity) of selected variables in CDWs and the ChCR. For this aim, the ChCR will be considered the reference. The SwissPedCancer team will perform these analyses, and all findings will be validated by a medical coder from the ChCR.

Accuracy checks will include:

1. Face validity: Assessment of clinical plausibility of data entries. For instance, any diagnosis not typically found in the pediatric population as first primary tumors (eg, breast or prostate cancer) or implausible age at diagnosis (eg, over 20 years) will be flagged for review.
2. Qualitative assessment of disagreements:
  - Cataloging and characterization: Discrepancies between datasets will be documented and described, including the proportion of observations affected and the types of inconsistencies.
  - Prioritization: Disagreements will be categorized as high, medium, or low priority based on two criteria: the percentage of affected observations and the clinical utility of the variable for the cancer registry. Variables with high disagreement and high relevance—such as diagnosis and treatment—will be prioritized for investigation.
3. Quantification of agreement: We will compute statistical measures such as Cohen κ, sensitivity, specificity, positive predictive value, and negative predictive value to evaluate the level of agreement between the two datasets. Cohen κ will be applied to assess concordance in the registration of high-priority variables, such as diagnostic coding.

#### Aim 4: Timeliness

We will evaluate the timeliness of childhood cancer registration, defined as the time interval between the date of cancer diagnosis (date of incidence) and the date of inclusion in official cancer statistics. We will assess data from 2016 to 2023, covering four years before and after the CRA. To provide a granular understanding of this timeline, we will decompose the overall interval into the following three distinct phases, as outlined in previous literature:

1. First registration: This period spans from the date when the cancer diagnosis is made and known (date of incidence) to the date when the case is first notified to and entered in the ChCR.
2. Final registration: The second interval begins with the initial recording of the case in the ChCR database and ends with the completion of internal quality assurance procedures. These procedures include the medical coding of the case by one medical coder and an independent cross-check by a second coder to ensure accuracy and consistency.
3. Publication phase: The final phase covers the time from the submission of aggregated registry data to the NACR until the publication of the data in the first statistical report. This interval reflects the broader dissemination of cancer statistics for public health monitoring and research use.

Timeliness will be reported as median time intervals with IQRs for each phase. Additionally, subgroup analyses will be conducted to explore differences in timeliness by age group, ICCC-3 main diagnostic group, and type of notifying institution, for example, diagnostic centers and pediatric oncology clinics.

#### Aim 5: Dataflow Processes

We will focus on improving the dataflow processes between CDWs and the ChCR, building upon the findings from aim 1 (comparability) and aim 2 (completeness). On the basis of identified discrepancies, missing data, and inconsistencies, we will propose targeted strategies to enhance efficiency, consistency, and automation of data transfers. These strategies include:

1. Harmonization and standardization of upstream data collection: For variables that exhibit low data quality or inconsistent registration across CDWs, we will recommend the implementation of more structured data entry formats. Harmonizing the standardized data extraction across Swiss pediatric oncology clinics and the ChCR will reduce variability and improve overall data quality.
2. Direct data integration: To minimize manual data entry, which is both labor-intensive and error prone, we will explore the feasibility of direct dataflows from CDWs to the ChCR. Automating this process would significantly reduce the burden on clinical and registry staff while improving data timeliness and accuracy.

In parallel, we will develop tools that complement the IARC and ENCR (European Network for Cancer Registries) check tools to support automated detection of data errors within the ChCR. Here, a custom R script or machine learning model will be designed to identify data errors systematically, defined as either missing variables or incomplete or inconsistent values for captured variables. These automated tools will flag problematic entries that can be addressed during subsequent data updates from pediatric oncology clinics. By incorporating automated quality checks into the data pipeline, the ChCR can proactively detect and resolve data integrity issues, leading to higher-quality datasets for analysis and reporting.

### Missing Data

Missing data in the ChCR and the pediatric oncology clinics’ clinical data will be described by variable, hospital, and incidence year. Subgroup analyses (eg, by cancer type, age group, or hospital) will be considered exploratory. To minimize overinterpretation, the number of subgroups will be limited, and findings will be interpreted using effect estimates and CIs rather than relying solely on *P*-values.

## Results

The project was funded in September 2022 and received ethics exemption in October 2023. Data extraction from participating hospitals and the ChCR is expected to commence in January 2026. Study results are anticipated to be available in Summer 2026.

## Discussion

### Main Findings

Completeness of registration of cancer cases—referred to as *case ascertainment*—is essential for drawing reliable conclusions about the development, progression, and treatment efficacy of the different cancer types. In Switzerland, case ascertainment in the ChCR is greater than 95% complete for children aged 0 to 15 years at diagnosis, as these patients are typically treated in specialized pediatric oncology clinics where cancer cases are routinely reported to the ChCR [40,41]. However, case ascertainment is lower for adolescents aged 16 to 19 years, whose care is fragmented across several pediatric and adult hospitals [40,41]. In 2010, 22% of individuals aged younger than 20 years and recorded in regional cancer registries were not registered in the ChCR. These missing individuals were mainly infants or adolescents [42]. Infants may die before being admitted to pediatric oncology clinics, making registration only possible via linkages with death certificate notifications of the Swiss Federal Statistical Office, whereas adolescents are often treated in adult oncology clinics with different registration procedures. Achieving complete case ascertainment across all age groups is critical to ensure accurate monitoring of pediatric cancer incidence, prevalence, survival, and quality of care. Comprehensive and reliable cancer registration enables timely cancer monitoring and supports public health planning [43]. Improvements in cancer registration—such as those proposed by SwissPedCancer—could enhance the ChCR’s data quality through the efficient and cost-effective transfer of CDW data and by using standardized and uniform procedures across pediatric oncology clinics.

Currently, there is no automated extraction of data from CDWs to the ChCR. Validation feedback loops are manual and cumbersome, resulting in delays in registering new patients and collecting follow-up data. Furthermore, it is redundant work, as the same data must be entered both in the primary record systems of the pediatric oncology clinics (from where it is automatically transferred into local CDWs) and again into the registry. The ChCR uses internationally standardized formats to code medical oncology data, which are often not used in primary record systems and associated CDWs, which further increases manual workload during data extraction and entry. The automated dataflows proposed by SwissPedCancer aim to reduce this manual effort while improving the completeness, accuracy, timeliness, and comparability of childhood cancer data in Switzerland. Ultimately, these enhancements aim to create a more robust, interoperable, and sustainable infrastructure for childhood cancer data in Switzerland—supporting accurate registration, timely analysis, and evidence-based decision-making.

### Strengths and Limitations

SwissPedCancer will include all nine pediatric oncology clinics in Switzerland, ensuring full national coverage. The comparison between CDW and ChCR data is a novel approach to assessing the completeness and quality of ChCR data from the nine pediatric oncology clinics. SwissPedCancer is the first project to describe the changes in quality indicators of cancer registration before and after the CRA came into force in 2020, which could have influenced completeness and timeliness of cancer registration.

One limitation of SwissPedCancer is that the COVID-19 pandemic started in 2020 which may have resulted in delays and interruptions to childhood cancer diagnosis and treatment [44]. However, in Switzerland, currently available evidence suggests that the effect of the COVID-19 pandemic on childhood cancer diagnosis and treatment was minimal, as in other high-income countries with healthcare systems that adapted quickly to maintain essential pediatric oncology services and avoid important delays in care [45]. Finally, as childhood cancer is a rare disease, which increases the chance of reidentification of the patients based solely on medical data, the procedures proposed in SwissPedCancer are designed to protect patients’ data by avoiding double data entry and minimizing the risk of data breach, which is a strength of this study.

### Outlook

SwissPedCancer presents a unique opportunity to assess the completeness and quality of cancer registration data in the ChCR compared to an external reference—CDWs from pediatric oncology clinics. It provides the opportunity to use existing data sources, which are rich in clinical details, for quality assurance. SwissPedCancer enables evaluation not only of case ascertainment and case completeness in the ChCR but also of data validity in pediatric oncology clinics and the ChCR.

Monitoring cancer at the population level has traditionally been labor-intensive, relying on manual record review and data entry by certified registrars [46]. Advances in information technology have expanded the availability of administrative databases, which are increasingly important for epidemiological research, quality assessment, and monitoring of disease outcomes [47]. CDWs, already in use at pediatric oncology clinics, have the potential to reduce the manual workload for registry staff and medical coders. While discrepancies may exist between pediatric oncology clinics and registry data—due to different objectives and methods of data collection—a shared objective remains: the accurate and complete data collection of cancer cases from the nine pediatric oncology clinics [48]. Data linkage and comparison between pediatric oncology clinics and the ChCR can generate new insights, broadening the potential impact of either data source when used alone. Finally, while the findings of this study are specific to childhood cancer, they are expected to inform the scalability of the approach toward other noncommunicable and communicable medical conditions, which may benefit from surveillance and clinical information system–enabled registries. By minimizing data handling, SwissPedCancer strengthens data privacy and supports efficient national data aggregation. The project’s standardized approach could serve as a model for other pediatric networks and broader national and international registry initiatives.

Assess adherence of cancer data to national and international standards: we will evaluate whether cancer data registered at the pediatric oncology clinics and at the ChCR align with the national and international guidelines for cancer registration and the standardization of practices.

## Supplementary material

10.2196/87007 Checklist 1 SPIRIT checklist.

10.2196/87007 Peer Review Report 1 Peer review report by the Swiss Personalized Health Network (SPHN)-Personalized Health and Related Technologies (PHRT) Review Committee (Switzerland).

## Abbreviations

BioMedIT: Biomedical Information Technology
CDW: clinical data warehouse
ChCR: Childhood Cancer Registry of Switzerland
CRA: Cancer Registration Act
CRO: Cancer Registration Ordinance
FAIR: Findable, Accessible, Interoperable, and Reusable
IARC: International Agency for Research on Cancer
ICCC-3: International Classification of Childhood Cancer–Third Edition
ICD-10: International Statistical Classification of Diseases, Tenth Revision
ICD-O-3: International Classification of Diseases for Oncology
NACR: National Agency for Cancer Registration
NDS: national data stream
PHRT: Personalized Health and Related Technologies
SNOMED CT: Systematized Nomenclature of Medicine Clinical Terms
SPHN: Swiss Personalized Health Network
SPIRIT: Standard Protocol Items: Recommendations for Interventional Trials
SwissPedHealth: Swiss Pediatric Personalized Research Network
