# Supplementary material for: Improving Registration and Dataflows Between Pediatric Oncology Clinics and the Childhood Cancer Registry of Switzerland: Protocol for SwissPedCancer Quality Assurance Study
Source: JMIR Res Protoc. 2026 Mar 10;15:e87007. doi: 10.2196/87007 (PMC12976597; doi:10.2196/87007)
Supplement: Peer Review Report 1 [file resprot-v15-e87007-s002.pdf]

SPHN – PHRT: Feedback and Questions & Answers session

Attendees:

Sarah Vermij (SPHN), Katrin Crameri (SIB, DCC), Daniel Vonder Mühll (PHRT), Francois Curtin (PHRT), David Harry (SPHN/PHRT?), Olivier Menzel (SPHN), Luregn Schlapbach (KISPI ZH), Matthias Baumgartner (KISPI ZH), Rebeca Mozun (KISPI ZH), Sean Froese (KISPI ZH), Julia Vogt (ETH ZH), Kelly Ormond (ETH ZH), Sandra Goetze (ETH ZH), Juliane Klatt (ETH ZH), Julia Bielicki (UKBB), Jaques Fellay (EPFL), Dylan Lawless (EPFL), Roger Lauener (KISPI SG), Eric Giannoni (CHUV), Phillip Latzin (Inselspital), Claudia Kuehni (ISPM Bern), Michaele Fux (ISPM Bern), Sandra Hunziker (ISPM Bern), Anne Tscherter (ISPM Bern), Fabiën Belle (ISPM Bern)

Excused:

Klara Posfay-Barbe (HUG), Effy Vayena (ETH Zurich), Martin Stocker (LUKS), Sven Schulzke (UKBB), Karsten Borgwardt (ETH ZH), Patrick Pedrioli (ETH ZH), Nicola Zamboni (ETH ZH), Jana Pachlopnik (KISPI ZH)

The goals of the meeting were to:

– Clarify requirements for full proposals
– Communicate feedback from outline proposal evaluation
– Discuss support and process for full application preparation
– Explain frame and boundary conditions, in particular use of PHRT funds
– Discuss lighthouse and nested projects including data generation

Feedback from outline proposal evaluation

The following are opinions expressed by expert panel members.
The selection decision was made by the whole panel.

Reviewer 1

- This a strong very well written proposal with strong governances and high social value. Key components of the project already exist (e.g. pediatric core dataset). There is good momentum for building a NDS and for ensuring its enduring value for partners and for personalized health research efforts in Switzerland and internationally. The consortium covers a great range of capable partners including non-academic institutions and biobanks, which gives the project a strong potential for instance, due to the inclusion of a variety of data types and expanding the analytical capabilities and potential research topics. They even have a non-funded partner in the USA. The project's aims and design will be furthered by the adoption of a governance framework that reflects the changing use of the digital technologies and takes account of the broader social context including the need for public and social consultation. These aspects will contribute to public acceptance of such initiatives, which will be critical to the long term impact and sustainability. The direct benefit to the consortium partners as well as social benefits, contributes to the proposal's strength. In the lighthouse project, a multi-omics pipeline will be established, for which they have identified 250 patients. The nested projects are good. The sustainability plan is feasible. Data governance is well addressed. The proposal demonstrates an understanding of the FAIR principles.
- Criticism: It is unclear whether technical aspects of all the potential data streams mentioned, such as access rights and agreements, will suffice to archive the desired grade of integration in the given time frame. It is unclear how the linkage between the different datasets will be implemented for the different nested projects given the variability of data sources and whether they can reach this within the NDS timeframe. The success of this NDS will depend on setting up the infrastructure to ensure clinical trial readiness to conduct Swiss wide studies including patient focused research. This a key outcome of the project. They main risk seems to be the capability and readiness of specific partners to contribute to the data stream. This needs to be assed and the project scaled accordingly. According to the feasibility report, certain partners are lacking in terms of ability to contribute to the NDS which creates a risk for full implementation in the given timeframe. The infrastructure may not be as far along as needed to complete the research projects.
- Other points: Stablishing a multi-omics pipeline to discover rare diseases will be of great significant social value. Achieving this is long term commitment and its success will depend on being able to build on the Swiss PedHealth data with high sensitive data and the development of methods and tools for research on personalized health. This worthy project may be overly ambitious given the timeframe. Either scaling back the aims or developing the project in stages, could reduce the scope for feasibility purposes, while setting the stage for success on a further stage. It is unclear whether interoperability between SPHN framework and Swiss PedData was already addressed during the SPHN infrastructure project.

Reviewer 2

- Strong project, strong integration of research, consortium is strong and multidisciplinary and builds on existing SPHN partnerships and solid infrastructure. The data stream is likely of sustained value in developing optimized procedures for pediatric diseases. The proposed work is not entirely novel, in that similar projects to analyze the genetics in children with extreme phenotypes have already begun elsewhere, e.g. in Australia, but outcomes from this work are nevertheless likely to provide interesting insights into personalized management of childhood diseases. No concerns about FAIR aspects, governance or structure. The clarity of the presentation of governance and management structure is considered a marker of high quality of project leadership.
- Criticism: in some participating hospitals, the required data flow has not been yet fully implemented. Data standardization and interoperability has not been fully aligned to the SPHN strategy. The feasibility report raised concerns about feasibility of establishing the multi-omics pipeline.

Reviewer 3

- The lighthouse project is exciting. The NDS has good long term prospects. The consortium is excellent with a good balance between basic researchers, data management experts and clinicians.
- Criticism: The wide range of participating institutions may complicate data integration. The small size of Switzerland may complicate recruitment for clinical research projects.

Reviewer 4

- Good integration of clinical and research data. The project aims to unify and harmonize Swiss wide pediatric clinical diagnostic and research omics data and includes multiple institutions. The consortium is highly qualified. The nested research projects are important. Contributions with high potential for scientific and patient impact.
- Criticism: probably a large effort is needed. The consortium lacks medical geneticists in the team. The lighthouse project is not new. The approach of rapid whole genome sequencing for critically ill patients in pediatrics and the evaluation of its utility for clinical decision making has been done in Australia, the US and UK for several years now with a significant number of citations. This includes the ELSI (ethical, legal, and social issues) part with interviews with health professionals and parents involved. In addition, this approach parallels with routine diagnostic rapid exome sequencing provided in medical clinical services in Switzerland and the current efforts allowing transition to whole genome sequencing in the near future. It is not clear why there are no collaborations envisaged with such medical genetic services in the proposal. It is not clear how and which resources for clinical genetics and genetic counselling in the clinics is provided. Genetic counselors are not yet a recognized profession in Switzerland. The timeline overall is very ambitious.

Feedback from DCC:

- Katrin Crameri explained that their focus is data interoperability, data standardization and compliance with the interoperability framework using semantic map technology as put forward by the SPHN steering board. In this regard they would need to work closely together with us to see if all our data providing institutions can provide data accordingly, and they are happy to help us and support us if we have specific questions during the elaboration of this proposal.
